# Patient Alerting Features in Implantable Defibrillators

**Published:** 2008-02-01

**Authors:** Dirk Vollmann, Markus Zabel, Johnson Francis

**Affiliations:** 1 Abteilung Kardiologie und Pneumologie, Herzzentrum, Universitatsmedizin Gottingen, Germany; 2 Calicut Medical College, Kerala, India

**Keywords:** Implantable cardioverter-defibrillator, Lead failure, Impedance measurement, Oversensing, Patient Alert

## Introduction

Implantable cardioverter defibrillators (ICD) are state of the art devices for the primary and secondary prevention of sudden cardiac death [1]. As a result, the use of ICDs has increased remarkably over the past years. Since they are life saving devices and because dysfunction can cause fatal pro-arrhythmia [2], monitoring of their proper functioning is vital for patient welfare.

To date, conventional ICD follow-up is in the form of device clinics where the ICD is interrogated and programmed periodically and the appropriate system function is ensured. Remote device monitoring has recently been introduced and may provide advantages especially for patients living further away from the implanting center [3]. Another important feature of current ICDs is the ability to monitor the device function and the patient clinical status, and to alert the patient if evidence for system dysfunction or adverse clinical events is found. This article gives an overview about patient alerting features of current ICDs.

## Alert features related to device function

Programming a ventricular fibrillation (VF) detection zone with shock therapy is essential for adequate ICD function. To ensure this condition, an alert may be triggered if VF detection and / or three or more VF therapies are programmed off by mistake.

ICD battery longevity is limited and capacitor charging times increase prior to battery depletion. Thus, it is important to exchange the device in time (when the recommended replacement time is reached). Currently available alert features warn the patient in case of low battery voltage and / or because of excessive capacitor charge time [4].

Lead-related adverse events occur in a small but significant proportion of ICD patients [4,5] and will probably become more frequent in the future since a growing amount of leads remains in place when generators are replaced for battery depletion. ICD lead failure may cause ineffective treatment during ventricular arrhythmia or inappropriate therapy due to oversensing of electrical noise [5,6]. Thus, early detection of ICD lead dysfunction, ideally prior to an adverse clinical event, is essential. Alert features that monitor lead impedance can enhance the early detection of ICD lead failure [4,5,7]. Devices with this feature deliver sub-threshold impulses on a daily basis to determine the impedance within the pace-sense or high voltage circuit of the ICD lead [5,8]. A very low lead impedance may indicate insulation failure, while a very high impedance may indicate conductor fracture. The upper and lower alert boundaries may be programmable, but typically a pacing lead impedance <200 or >2,500 to 3000 Ω and high-voltage lead impedance <10 to 20 or >200 Ω is considered abnormal and will trigger an alert.

Furthermore, an alert may be triggered if anywhere from one to six high voltage shocks occur during one single episode or if all device therapies within one tachycardia detection zone are delivered. Both of these alerts may indicate device dysfunction (e.g., ventricular oversensing) or a clinical problem like an incessant VT or atrial fibrillation with rapid atrioventricular conduction.

## Alert features related to patient clinical status

Most ICD patients are also at risk for clinical events other than ventricular tachyarrhythmias. The so-called OptiVol™ alert feature is based on intrathoracic impedance monitoring and has been introduced in an effort to enhance the early detection of  decompensating chronic heart failure (CHF) [9]. Observational data suggest that this alert feature can be integrated into the ambulatory management of  CHF patients [9].

Other alert features are related to the occurrence of atrial tachyarrhythmias. An alert that warns the patient if the daily atrial tachyarrhythmia burden exceeds a programmable value may help to prevent thrombembolic events [11]. Another alert feature is triggered if the average ventricular rate during an atrial tachyarrhythmia episode crosses a programmable threshold, thereby preventing high ventricular rates that may lead to CHF decompensation.

## Limitations of current alert features and future perspective

There is a broad variation in the availability of alert features between ICD models from different manufacturers. Most of the existing alert features are available in recent Medtronic ICDs. In these devices, the patient can be alerted by an audible signal, occurring once daily at a programmable time. The limitation of using an acoustic signal is that some patients may not hear the alert, while other subjects may confuse environmental noise with the device alert tone. This can result in "phantom alerts" that cannot be verified upon ICD interrogation. To overcome these limitations, an external device (available in Europe) that provides wireless communication with the implanted ICD can be handed out to these subjects. In case of an alert event, patients are then visually warned (signal light on). In recent devices from St. Jude Medical, vibration of the ICD is used to alert the patient of potential device dysfunction. One limitation of acoustic, visual and tactile alerts is that the patient has to contact the physician first to allow an intervention. Recent devices offer automatic remote transmission of alert events and associated diagnostic data, which may allow a more rapid event verification and intervention by the physician.

Despite obvious advantages, patient alerting features do not yet substitute for regular follow-up visits. In particular, current alert algorithms have only a limited sensitivity to detect lead-related problems [4,5]. Lead dislocation may not be detected because pacing and sensing thresholds are not monitored. Impending ICD lead failure is often not detected sufficiently early, because sporadic measurements of impedance (once daily) are unlikely to reveal unusual findings if the structural lead defect is discrete at first and electrical integrity is lost only for brief moments (e.g. during arm movement). Continuous monitoring of lead integrity can be achieved by the Sensing Integrity Counter (SIC, Medtronic), which is a cumulative count of very short ventricular senses intervals that indicate oversensing of electrical noise. Retrospective data suggest that the combined use of SIC and impedance monitoring would  result in very high sensitivity for ICD lead failure detection [5,12]. Unfortunately, however, this feature has not yet been integrated into a patient alert algorithm.

Alert features that monitor the clinical status of ICD patients, e.g. for impending CHF decompensation or the onset of atrial tachyarrhythmias, are exciting new diagnostic tools that may improve patient management. However, it is not yet clear how these diagnostic data should be integrated into routine clinical practice, and if hard endpoints such as hospitalizations or mortality can be reduced. Large randomized controlled trials are on the way to answer these questions.
